# The protective effect of traditional Chinese medicine Jinteng Qingbi granules on rats with rheumatoid arthritis

**DOI:** 10.3389/fphar.2024.1327647

**Published:** 2024-03-13

**Authors:** Yingying Wan, Wenting Sun, Jiaxi Yang, Haonan Wang, Wenqian Wang, Wanting Ye, Guoliang Cheng, Bing Li, Jianxun Ren, Qiuai Kou

**Affiliations:** ^1^ Dongfang Hospital, Beijing University of Chinese Medicine, Beijing, China; ^2^ China Academy of Chinese Medical Sciences, Xiyuan Hospital, Beijing, China; ^3^ Shaanxi Provincial Hospital of Traditional Chinese Medicine, Xian, China; ^4^ The Fourth People’s Hospital of Jinan, Jinan, China; ^5^ Key Laboratory of Generic Pharmaceutical Technology for Chinese Medicine, Lunan Pharmaceutical Group Co. Ltd, Linyi, China

**Keywords:** Jinteng Qingbi granules, rheumatoid arthritis, synovial tissue proteomics, NF-κB pathway, serum metabolomics

## Abstract

**Introduction:** Jinteng Qingbi granules (JTQBG), a traditional Chinese medicine formulation, are widely used for the treatment of rheumatoid arthritis (RA) due to their satisfactory therapeutic efficacy. However, the underlying mechanism of action remains unclear. This study aims to investigate the protective effects of JTQBG against RA and elucidates its potential molecular mechanisms.

**Methods:** A collagen-induced arthritis (CIA) rat model was utilized, and JTQBG (1.25, 2.5, 5 g/kg/day) or methotrexate (MTX, 1 mg/kg/week) was orally administered. The rats’ weight, arthritis index (AI), and paw volume were measured weekly. Synovial hyperplasia of the joints was detected using a small animal ultrasound imaging system. Joint destruction was assessed using an X-ray imaging system. Histopathological examinations were performed using hematoxylin-eosin (H&E), Saffron-O and fast green staining. Serum inflammatory cytokines were detected using ELISA. Furthermore, 4D label-free quantitative proteomics of synovial tissues and non-targeted metabolomics of blood serum were conducted to analyze the molecular mechanisms.

**Results:** JTQBG exerted a significant therapeutic effect on CIA rats by reducing inflammatory cell infiltration, synovial hyperplasia, cartilage erosion, and bone destruction. It also decreased the spleen index, inhibited hyperplasia of the white pulp, and decreased the serum levels of IL-1β and IL-18. Proteomics analysis identified 367 differentially expressed proteins (DEPs) between the Model and Normal groups, and 71 DEPs between the JTQBG and Model groups. These DEPs were significantly enriched in the NF-κB pathway. 11 DEPs were significantly reversed after treatment with JTQBG. Western blot results further validated the expression levels of Nfkb1, Pdk1, and Pecam1, and analyzed the expression levels of p-IKK, p-IκBα, and IκBα. The therapeutic efficacy of JTQBG was partly attributed to the suppression of the NF-κB pathway in synovial tissues. Serum metabolomics identified 17 potential biomarkers for JTQBG treatment of CIA rats, which were closely related to Alanine, aspartate and glutamate metabolism, Tryptophan metabolism, Ascorbate and aldarate metabolism, Arginine metabolism, and Inositol phosphate metabolism.

**Conclusion:** Our findings demonstrated that JTQBG was effective against RA by alleviating synovial inflammation, synovial hyperplasia, and joint destruction. The anti-RA properties of JTQBG were likely attributed to the inhibition of the NF-κB pathway and the regulation of serum metabolite disorders.

## Introduction

Rheumatoid arthritis (RA) is a systemic autoimmune and inflammatory disorder with high incidence around the world (Tobón et al., 2010). In RA, immune cells and activated macrophages infiltrate synovial tissues, which results in persistent synovial inflammation, pannus and the progressive destruction of bone and cartilage and eventually causes symptoms of musculoskeletal pain, swelling and stiffness (Sparks, 2019; Auréal et al., 2020). The secretion of inflammatory cytokines from synovial fibroblasts, macrophages and chondrocytes results in the progression of RA (Brzustewicz and Bryl, 2015; Kim et al., 2018). The continuous inflammation of synovial tissue causes an imbalance of proliferation and apoptosis of fibroblast-like synoviocytes, followed by the degradation of the collagen cartilage matrix (Noss and Brenner, 2008; Yoshitomi, 2019).

Therefore, the synovial tissue, as the main pathological site of RA, attenuates synovial inflammation and synovial hyperplasia and plays an essential role in the treatment of RA (Ren et al., 2021). To our knowledge, nonsteroidal anti-inflammatory drugs, disease-modifying antirheumatic drugs, glucocorticoids and biological agents have been commonly used to improve symptoms and disease progression. Nevertheless, these drugs have failed to produce satisfactory pharmacological effects along with severe side effects such as immunodeficiency, gastrointestinal disorders and bodily fluid disorders (Liu et al., 2022a). Traditional Chinese Medicine has been recognized as a potential pharmacological target in anti-arthritis therapy in Asian countries by reducing inflammatory and synovial hyperplasia and inhibiting bone and cartilage destruction (Li et al., 2019; Chen et al., 2020; Xie et al., 2021).

In recent years, proteomics and metabolomics techniques have been used to detect synovial tissue, blood and other biological samples of RA (Gobezie et al., 2006; Park et al., 2015). Relevant studies have shown that the Protein and metabolic pathway of RA has changed, and it may participate in the pathogenesis of RA by promoting inflammation and the immune response (Zhou et al., 2016; Takahashi et al., 2017) The differentially expressed proteins and metabolites could be biomarkers of RA (Song and Lin, 2017; Mun et al., 2019).

JTQBG, a traditional Chinese medicine formulation, has been approved by the China Food and Drug Administration. It consists of eleven kinds of botanical drugs, as shown in Table 1. JTQBG is derived from a traditional well-known formula “Si Miao Yong’an decoction” originated in the record in New Compilation of Effective Recipes and is used to treat blocking collaterals in “Bi syndrome”. Previous studies have revealed that JTQBG was beneficial for RA and could improve clinical symptoms without any adverse events (Sun et al., 2018; Hu, 2020). However, the anti-RA mechanisms of JTQBG have not been validated. In this study, we used CIA model to assess the therapeutic effect of JTQBG and explored the anti-RA mechanism through synovial proteomics.

**TABLE 1:** Formulation of JTQBG.

Botanical or zoological name	Traditional name in Chinese	Produced from	Basic phytochemical metabolites	Crude drug(g) in daily prescription
*Lonicera japonica* Thunb. [Caprifoliaceae]	Jinyinhua	Dried flower bud	chlorogenic acid, isochlorogenic acid A, 4-caffeoylquinic acid et al	15
Rehmannia glutinosa (Gaertn.) DC. [Orobanchaceae]	Dihuang	Dried root	Rehmannioside D, catalpol et al	15
Scrophularia ningpoensis Hemsl. [Scrophulariaceae]	Xuanshen	Dried root	Harpagoside, harpagide, maltose et al	7.5
Paeonia lactiflora Pall. [Paeoniaceae]	Baishao	Dried root	Paeoniflori, albiflorin et al	15
Cremastra appendiculata (D.Don) Makino [Orchidaceae]	Shancigu	Dried pseudobulb	Betaine, berberine et al	5
Angelica sinensis (Oliv.) Diels [Apiaceae]	Danggui	Dried root	Angelicide, ferulic acid et al	7.5
Scleromitrion diffusum (Willd.) R.J.Wang [Rubiaceae]	Baihuasheshecao	Dried herba	Geniposidic acid, quercetin et al	10
Glycyrrhiza glabra L. [Fabaceae]	Gancao	Dried root and rhizome	Liquiritin, isoliquiritigenin et al	5
Microsorum scolopendria (Burm.f.) Copel. [Polypodiaceae]	Wugong	Dried body		1
Sinomenium acutum (Thunb.) Rehder and E.H.Wilson [Menispermaceae]	Qingfengteng	Dried rattan	Sinomenine, acutumine, et al	15
Pyrola calliantha H. Andres [Ericaceae]	Luxiancao	Dried herba	Monotropein, isohomoarbutin, et al	10

## Materials and methods

### Animals

Male Sprague‒Dawley rats (180–200 g) were purchased from Beijing Weitong Lihua Laboratory Animal Technology Co., Ltd. (certificate number: SCXK (JING)20210006) and housed in standard laboratory conditions with food and water freely available. The animal experiment was approved by the Animal Experiment Ethics Committee of Xiyuan Hospital, China Academy of Chinese Medical Sciences, Beijing, China (approval number: 2021XLC045-2) in accordance with the National Institutes of Health (NIH) Guidelines for the Care and Use of Laboratory Animals.

### Chemicals and reagents

Methotrexate (MTX) was purchased from Shanghai Shangyao Xinyi Pharmaceutical Co., Ltd. (Shanghai, China). Bovine Type II Collagen-Solution was purchased from Chondrex. Freund’s complete adjuvant was purchased from Sigma‒Aldrich.

### UHPLC-MS/MS conditions of phytochemical analysis

JTQBG was provided by Lunan Pharmaceutical Co., Ltd. (Linyi, China; Approval no. Z20123065; Batch number: 28210021). Chromatographic separation was performed on the Dionex UltiMate™ 3000 (Thermo-Fisher Scientific, USA) and a paired UPLC HSS T3 column (1.8 μm, 2.1 × 100 mm, Waters, USA). The chromatographic conditions were displayed as follows: flow rate of 0.3 mL/min, column temperature of 30°C, 0.1% formic acid solution (phase A) and acetonitrile (phase B). MS condition was carried out on a Thermo-Fisher Q-Exactive™ (Thermo-Fisher Scientific, USA). Full MS/dd-MS^2^ was performed on a heated electrospray ionization (HESI) including the positive and negative modes at the mass range of 100–1200 m/z. Other parameter settings were as follows: capillary temperature of 320°C, vaporizer temperature of 300°C, spray voltage of 3.7/3.5 kV (ESI+/ESI-), sheath gas pressure of 30 psi, and auxiliary gas pressure of 10 psi. Additionally, the content of the main metabolites of JTQBG was analyzed using the ACQUITY UPLC I-CLASS (Waters, USA) and XEVO TQ-S Micro (Waters, USA).

### CIA establishment and treatment

According to a previous study (Deng Y. et al., 2020; Liu C. et al., 2021), bovine type II collagen (50 mg) was dissolved in 0.01 mol/L acetic acid (25 mL) at 4°C and diluted in an equal volume of complete Freund’s adjuvant to a final concentration of 1.0 mg/mL. For the CIA model, 0.5 mL of CII emulsion was subcutaneously injected at the base of the rat tail (0.1 mL), three points on the back (0.1 mL x 3) and the right hind paw (0.1 mL) on day 0. A booster immunization (0.2 mL) was given at the base of the rat tail on day 7, avoiding the primary injection site. Rats in the Normal group (**Normal**, *n* = 10) were injected with the same volume of saline at the corresponding positions. The rats with successful induction of CIA were randomly divided into five groups (*n* = 10): the Model group (Model), JTQBG low-dose group (JTQBG-L), JTQBG medium-dose group (JTQBG-M), JTQBG high-dose group (JTQBG-H), and positive control MTX group (MTX). The animal dose in our study was converted based on the body surface area (BSA) normalization method, as was reported by the previous studies (Xu, et al., 2002; Yao et al., 2023). From day 7 to day 42, rats in the JTQBG groups (JTQBG-L, JTQBG-M and JTQBG-H) were orally administered JTQBG at concentrations of 1.25 g/kg, 2.5 g/kg, and 5 g/kg, respectively, once a day, and in the MTX group, MTX (1 mg/kg) was administered once a week. All rats in the Normal and Model groups received an equal volume of saline.

### Clinical evaluation of CIA

Two independent observers with no knowledge of the treatment protocol evaluated the severity of RA. Beginning on the 7th day after the first immunization, the body weight, paw volume, and arthritis index (AI) of the rats were evaluated every 7 days. The AI of CIA rats was scored as follows: 0 = “no swelling or redness,” 1 = “phalanx joints slightly swollen and redness,” 2 = “phalanx joints and paw swelling and redness,” 3 = “phalanx joints and paw swelling and redness except the ankle joints,” and 4 = “whole paws and ankle joint swelling and redness.” All paws were measured, and the maximum value of each rat was 16 (Shi et al., 2022).Synovial hyperplasia of joint was detected by a small animal ultrasound imaging system. Joint damage of joint was detected by An X-ray imaging system.

### Histopathological analysis

The knee joints and spleens were fixed with 10% paraformaldehyde. Then, the knee joints were decalcified by a decalcification solution. After dehydration with alcohol, vitrification with xylene, embedding in paraffin, and sectioning, the sections were stained with hematoxylin-eosin (H&E) to observe pathological changes in the knee joints and spleens. The histological score of the knee joints was evaluated blindly as described previously (Liu et al., 2019). In addition, sections were stained with Safranin O and Fast Green for cartilage observation, and cartilage damage was evaluated according to the criteria reported by Mukai et al. (Mukai et al., 2015).

### Detection of serum inflammatory cytokines

Commercial ELISA kits were used to examine the levels of IL-1β and IL-18 according to the manufacturer’s instructions. The content of inflammatory cytokines in the samples was calculated by standard curve.

### Proteomics analysis

According to previous studies (Lin et al., 2021; Shu et al., 2021; Zhu et al., 2021), protein was extracted from fresh synovial tissues and quantified using a BCA protein assay kit. A total of 200 μg of extracted protein was incorporated into 30 μL of SDT buffer. The protein suspensions were digested with trypsin, and the resulting peptides were collected. Twenty micrograms of protein was mixed with 5x loading buffer, boiled for 10 min and separated on a 12.5% SDS‒PAGE gel. Protein from synovial tissues was analysed through LC‒MS/MS on a Q Exactive Plus mass spectrometer (Thermo Scientific). The identification and quantitation of protein were performed using MaxQuant 1.5.3.17 software. Protein was significantly changed if fold changes >1.5, <1:1.5, and *p* < 0.05, which were considered DEPs. Next, we performed a series of bioinformatics analyses, including cluster analysis, subcellular localization, and gene ontology (GO) analyses. The cluster analysis was performed by the genescloud tools (https://www.genescloud.cn). More importantly, we blasted the studied proteins against the online Kyoto Encyclopedia of Genes and Genomes (KEGG) database.

### Western blotting

Western blotting was performed as previously described (Liu et al., 2022b). Briefly, total proteins were extracted from synovial tissues using a cell lysis buffer supplemented with proteinase inhibitor on ice for 1 h. The protein concentrations were measured using a BCA protein assay kit (Beyotime). The extracted protein (30 μg per sample) was separated on 4%–12% polyacrylamide gels (Thermo Fisher Scientific), electroblotted onto a nitrocellulose membrane, and probed with primary antibody followed by incubation with horseradish peroxidase (HRP)-coupled secondary antibody against the primary antibody. The primary antibodies used in this study were rabbit anti-phospho-Nfkb1 (1:1000; Cell Signaling Technology; #4806), anti-Nfkb1 (1:500; Abcam; ab28849), anti-Pecam1 (1:1000; Abcam; ab281583), anti-Pdk1 (1:2000; Abcam; ab207450), anti-phospho-IKK (1:1000; Cell Signaling Technology; #2697), anti-phospho-IκBα (1:10000; Abcam; ab133462), and IκBα (1:1000; Abcam; ab32518). After incubation with the appropriate HRP-coupled secondary antibodies (Proteintech), visualization of the protein bands was performed by incubation with SuperSignal West Pico Chemiluminescent Substrate (Thermo Fisher) and then documented using the ChemiDoc™ MP System (Bio-Rad).

### Metabolomics analysis

Additionally, serum samples were collected for metabolomics analysis, followed by metabolite extraction. For LC-MS analysis, all samples were dissolved in a 1:1 mixture of acetonitrile and water and transferred to LC vials. The extracts were analyzed using a Sciex TripleTOF 6600 quadrupole time-of-flight mass spectrometer (Sciex, USA) coupled with hydrophilic interaction chromatography. In MS acquisition, the instrument was set to acquire data in the m/z range of 60–1000 Da, with a scanning cumulative time of 0.20 s per spectra for TOF MS. In MS/MS acquisition, the instrument was set to acquire data in the m/z range of 25–1000 Da, with a scanning cumulative time of 0.05 s per spectra for the product ions. Pareto-scaled principal component analysis (PCA) was conducted. Our studies revealed changes in serum metabolites, differentially expressed metabolites (DEMs), and enrichment of metabolic pathways (León et al., 2013; Benton et al., 2015).

### Statistical analysis

All quantitative data are expressed as the mean ± standard deviation (SD). Student’s t-test or one-way analysis of variance (one-way ANOVA) followed by Scheffe’s *post hoc* test was performed for normally distributed data, and the Kruskal-Wallis test was performed for non-normal data using SPSS version 22.0 (SPSS, Chicago, IL, USA). Statistical significance was considered as *p* < 0.05 in our study.

## Results

### Identification of the metabolites in JTQBG

**FIGURE 1:**
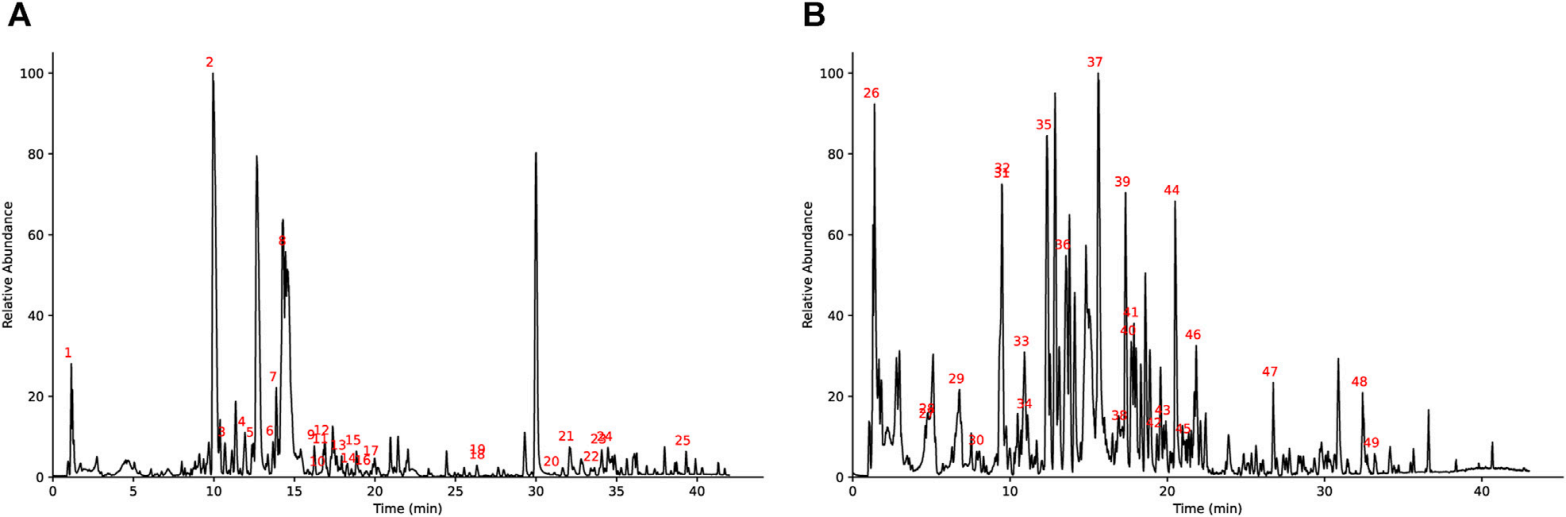
Base peak ion chromatogram of JTQBG in positive ion mode **(A)** and negative ion mode **(B)**.

Figure 1 shows the base peak intensity chromatogram (BPI) of JTQBG. The complete details of all the metabolites identified in both positive and negative ion modes for JTQBG can be found in Supplementary Material S1, S2. Table 2 provides a list of 49 main metabolites along with their basic formulae. Furthermore, the quantitative analysis of JTQBG revealed that the high contents were observed for paeoniflorin (1018.12 μg/g), sinomenine (560.08 μg/g), chlorogenic acid (294.27 μg/g), isochlorogenic acid A (205.40 μg/g), ferulic acid (121.60 μg/g), monotropein (111.00 μg/g), harpagide (94.21 μg/g), liquiritin (90.44 μg/g), rehmannioside D (68.66 μg/g), galuteolin (40.00 μg/g), betaine (34.67 μg/g), and quercetin (4.94 μg/g).

**TABLE 2:** Details of 49 main metabolites in JTQBG.

Number	Main metabolites	m/z	Retention time (min)	Adducts	Formula	Peak area
1	betaine	118.09	1.18	M + H	C5H11NO2	1647343.72
2	sinomenine	330.17	9.94	M + H, M + Na	C19H23NO4	683794866.50
3	acutumine	398.14	10.68	M + H	C19H24ClNO6	38850361.76
4	4-caffeoylquinic acid	355.10	11.91	M + H-H2O, M + H, M + Na, M + K	C16H18O9	35233672.73
5	caffeic acid	163.04	12.44	M + H-2H2O, M + H-H2O, M + H	C9H8O4	11177331.13
6	albiflorin	481.17	13.67	M + H, M + NH4, M + Na, M + K	C23H28O11	77104617.01
7	magnoflorine	342.17	13.88	M+	C20H24NO4^+^	82927379.33
8	4′-o-methylglabridin	356.19	14.45	M + NH4	C21H22O4	317062697.30
9	gancaonin x	356.19	16.23	M + NH4	C21H22O4	31787476.73
10	quercetin-3-o-[β-d-glucopyranosyl-(1–2)-β-d-galactopyranoside]	627.16	16.52	M + H, M + Na	C27H30O17	8140904.18
11	ferulic acid	177.05	16.72	M + H-H2O, M + H	C10H10O4	4311206.46
12	(−)-8-oxotetrahydrothalifendine	322.11	16.80	M + H-H2O	C19H17NO5	26704158.54
13	l-phenylalaninosecologanin	538.23	17.92	M + H	C26H35NO11	34098029.52
14	3,5-Dicaffeoylquinic acid	499.12	18.53	M + H-H2O, M + H, M + Na	C25H24O12	27312764.42
15	sinomendine	338.14	18.84	M + H	C20H19NO4	26283186.48
16	quercetin	303.05	19.38	M + H	C15H10O7	4975196.40
17	berberine	336.12	19.86	M+	C20H18NO4+	13165455.46
18	isoliquiritigenin	257.08	26.29	M + H	C15H12O4	2214406.68
19	harpagoside	517.17	26.34	M + Na, M + K	C24H30O11	22194228.87
20	licoricesaponine g 2	839.40	31.13	M + H-H2O, M + H, M + Na	C42H62O17	48576944.39
21	18 alpha-glycyrrhetinic acid	453.34	32.08	M + H-2H2O, M + H-H2O, M + H	C30H46O4	101988357.10
22	uralsaponin B	845.39	33.56	M + NH4, M + Na, M + K	C42H62O16	28857996.62
23	angelicide	403.19	34.08	M + H-H2O, M + H, M + NH4, M + Na	C24H28O4	75803510.75
24	levistolid A	403.19	34.46	M + H, M + NH4, M + Na	C24H28O4	89263012.37
25	1-monolinolein	377.27	39.31	M + H-H2O, M + H, M + NH4, M + Na, M + K	C21H38O4	23898870.63
26	maltose	341.11	1.43	M-H, M + FA-H	C12H22O11	49770501.52
27	catalpol	407.12	4.61	M-H, M + FA-H	C15H22O10	15859881.46
28	monotropein	389.11	4.61	M-H	C16H22O11	7056556.27
29	isohomoarbutin	331.10	6.79	M + FA-H	C13H18O7	18758641.91
30	rehmannioside D	731.23	7.98	M-H, M + FA-H	C27H42O20	3639888.68
31	isochlorogenic acid	353.09	9.48	M-H	C16H18O9	32676887.92
32	harpagide	409.13	9.57	M-H, M + FA-H	C15H24O10	10440175.44
33	oxypaeoniflorin	495.15	11.01	M-H	C23H28O12	453013.89
34	catechin	289.07	11.21	M-H	C15H14O6	54698.22
35	chlorogenic acid	353.09	12.35	M-H, M + Na-2H	C16H18O9	33539784.96
36	geniposidic acid	373.11	13.55	M-H	C16H22O10	28762988.81
37	paeoniflorin	525.16	15.61	M-H, M + FA-H	C23H28O11	124111604.30
38	7-epi-vogeloside	433.13	17.17	M-H, M + FA-H	C17H24O10	18704941.89
39	deacetyl asperulosidic acid methyl ester	403.12	17.35	M-H, M + FA-H	C17H24O11	26686675.48
40	liquiritin	417.12	17.71	M-H	C21H22O9	16514559.91
41	6-o-e-p-coumaroyl scandoside methyl ester	549.16	17.88	M-H	C26H30O13	37580663.19
42	cinaroside	447.09	19.34	M-H	C21H20O11	4390034.33
43	hyperoside	463.09	19.82	M-H	C21H20O12	3076929.92
44	iso chlorogenic acid a	515.12	20.49	M-H, M + Na-2H	C25H24O12	49420747.60
45	centauroside	803.26	21.21	M-H, M + FA-H	C34H46O19	12061799.62
46	liquiritigenin	255.07	21.88	M-H	C15H12O4	3306799.99
47	8-o-feruloylharpagide	539.18	26.74	M-H, M + Na-2H	C25H32O13	11454576.98
48	glycyrrhizic acid	821.40	32.42	M-H	C42H62O16	84972591.73
49	licoricesaponine k2	821.40	33.18	M-H, M + Na-2H	C42H62O16	30358541.99

### JTQBG alleviated the symptoms of arthritis in CIA rats

**FIGURE 2:**
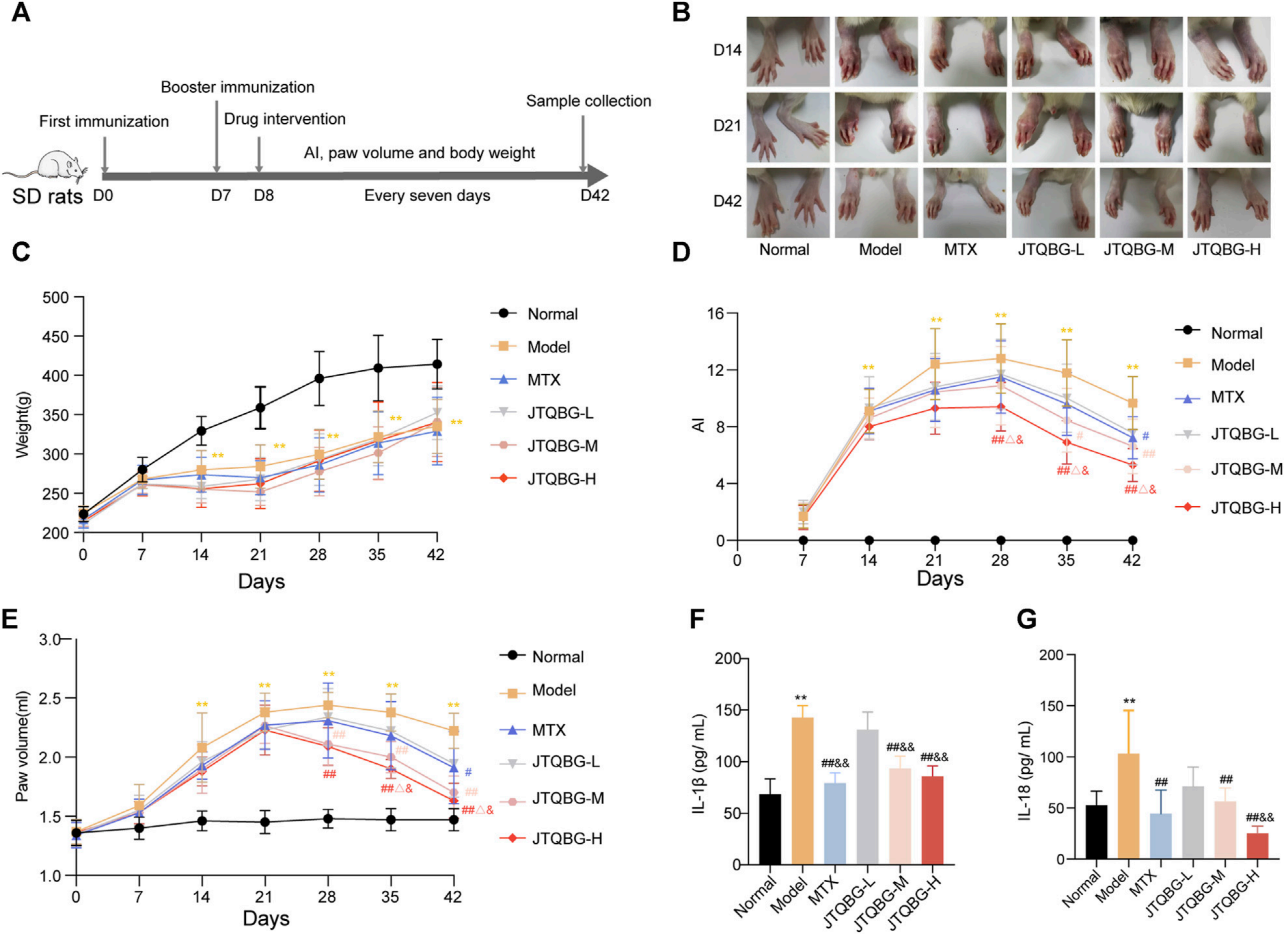
Effects of JTQBG on the pathological progress of CIA rats. **(A)** Experimental procedure for evaluating the anti-arthritic effect of JTQBG. **(B)** Effects of different doses of JTQBG and MTX on paw inflammation at different stages. **(C)** Average body weight per group was measured. The arthritis index (AI) **(D)** and paw volume **(E)** were measured and calculated. **(F)** The ELISA assay of IL-1β in serum. **(G)** The ELISA assay of IL-18 in serum. Values are expressed as the mean ± SD. *p < 0.05, **p < 0.01 compared with the Normal group; #*p* < 0.05, ##*p* < 0.01 compared with the Model group; △*p* < 0.05, △△*p* < 0.01 compared with the MTX group; &*p* < 0.05, and&*p* < 0.01 compared with the JTQBG-L group.

The anti-arthritic effect of JTQBG was investigated by measuring various parameters related to the symptoms of RA rats, such as arthritis index, paw volume and body weight, in accordance with the experimental procedure, as shown in Figure 2A. The paw swelling of rats was observed in different immune periods. In comparison to the Normal group, the Model group exhibited paw swelling on day 14, peaked level on day 21 and then gradually decreased, while different doses of JTQBG and MTX alleviated the degree of paw swelling in rats (Figure 2B). The weight gain rate of the Model group became slower than that of the Normal group after the second immunization. Nevertheless, there was no significant difference with respect to weight after JTQBG and MTX treatment (Figure 2C). On day 14 after immunization, the AI and paw volume in the Model group were significantly increased compared with those in the Normal group (*p* < 0.01). Compared with the Model group, the JTQBG-H, JTQBG-M and MTX groups showed reduced AI and paw volume (Figures 2D, E). Interestingly, these improvements were more pronounced in the JTQBG-H group, with a remarkable decrease in AI and paw volume (*p* < 0.01) on day 28 after immunization, and significantly lower than those in the MTX and JTQBG-L groups in AI (*p* < 0.05) on day 28 and paw volume (*p* < 0.05) on day 35 after immunization. The JTQBG-M group had significantly reduced AI (*p* < 0.05) on day 35 and paw volume (*p* < 0.01) on day 28 after immunization, while the MTX group also had markedly reduced AI and paw volume (*p* < 0.05) on day 42 after immunization. Moreover, we found that the serum levels of IL-1β and IL-18 were significantly increased in Model group compared with Normal group (*p* < 0.01) (Figures 2F,G). JTQBG-H, JTQBG-M and MTX groups significantly reduced the level of IL-1β and IL-18.

### JTQBG improved synovial hyperplasia in CIA rats

**FIGURE 3:**
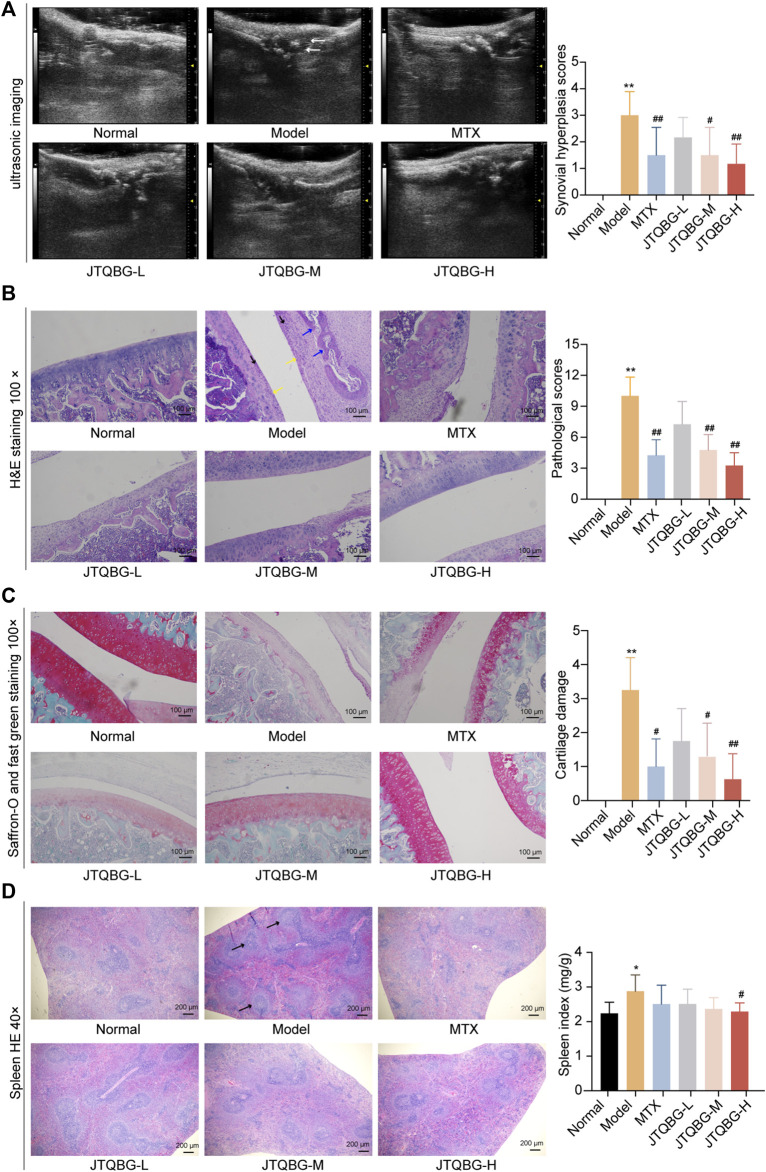
Therapeutic effects of JTQBG on the joint and spleen. **(A)** Representative images of joint ultrasonic imaging and synovial hyperplasia score. Synovial hyperplasia was marked by white arrows. **(B)** Representative images of H&E staining of knee joints and pathological scores. Inflammatory cells were marked by black arrows. Synovial hyperplasia was marked by yellow arrows. Cartilage erosion was marked by blue arrows. Scale bar: 100 μm. **(C)** Representative images of Saffron-O and fast green staining of knee joints and cartilage damage scores. Scale bar: 100 μm. **(D)** Representative images of H&E staining in spleens and spleen index. White pulp was marked by black arrows. Scale bar: 200 μm *p < 0.05, **p < 0.01, compared with the Normal group; #*p* < 0.05, ##*p* < 0.01, compared with the model group. UBM: Ultrasound Biomicroscopy.

To further evaluate the effect of JTQBG on synovial hyperplasia in RA rats, ultrasound examination images of small animals were performed, which revealed that the skin of Normal group shown a constant thickness with homogeneous texture. Compared with the Normal group, the distance between the skin surface and bone limits of the Model group was greatly enlarged due to the infiltration of inflammatory edema tissue, and the echographic appearance was inhomogeneous. Following the treatment, JTQBG groups and MTX group were improved (Figure 3A). We also scored synovial hyperplasia of the joint in each group. The results showed severe synovial hyperplasia in the Model group compared with the Normal group (*p* < 0.01). However, synovial hyperplasia in the JTQBG-H or MTX group (*p* < 0.01) exhibited obvious mitigation compared with that of the Model group. Collectively, these results indicated that JTQBG alleviated the severity of arthritis in CIA rats.

### JTQBG improved pathological phenomena and joint damage in CIA rats

To determine the protective effects of JTQBG against RA, histopathological changes in the knee joints were evaluated by H&E staining. As shown in Figure 3B and Supplementary Material S3A, the articular surface of normal rats was smooth and complete, and the joint space was uniform. Moreover, the synovial cell layer was neatly located on the lateral side of the cartilage. Compared with normal rats, the synovial cells were significantly hyperplastic and disordered along with excessive inflammatory cell infiltration and cartilage erosion in the Model group rats. Nevertheless, these pathological phenomena were significantly improved with respect to inflammatory cell infiltration, synovial proliferation, and cartilage and bone erosion (*p* < 0.01) after treatment with high or medium doses of JTQBG or MTX. Next, Saffron-O and fast green staining were used to investigate the changes in articular cartilage. The results showed severe cartilage damage in the Model group compared with the Normal group (*p* < 0.01). However, cartilage damage in the JTQBG-H (*p* < 0.01), JTQBG-M or MTX group (*p* < 0.05) exhibited obvious improvement compared with that in the Model group (Figure 3C and Supplementary Material S3B). JTQBG could alleviate lesions of the knee joints and synovial tissues caused by RA. The spleen index and histopathological changes in the spleen were also investigated (Figure 3D). The observation of the pathological sections showed that the splenic white pulp of normal rats was crossed by the central artery and surrounded by a marginal region. Compared with the Normal group, the deformed structure and the proliferation of white pulp were distinctly observed in the spleens of the Model group. After treatment with JTQBG or MTX, the white pulp deformations tended to improve. The spleens of the rats were weighed to calculate the organ index. Compared with the Normal group, the spleen index in the Model group increased significantly (*p* < 0.05). In contrast, the spleen index in the JTQBG-H group decreased significantly (*p* < 0.05) compared with that in the Model group. These results indicated that JTQBG has an immunosuppressive effect, thereby reducing spleen swelling.

Further, we conducted X-ray imaging to evaluate the effect of JTQBG on joint damage in CIA rats. As shown in Supplementary Material S4, compared to Normal group, the CIA rats showed severe joint damage, with blurred articular surface, enlarged joints, substantial bone erosion, narrowed joint space, and swelling of joint soft tissue. However, after the treatment of JTQBG, the sign of joint damage was significantly alleviated.

### JTQBG affected the expression and function of synovial proteins

**FIGURE 4:**
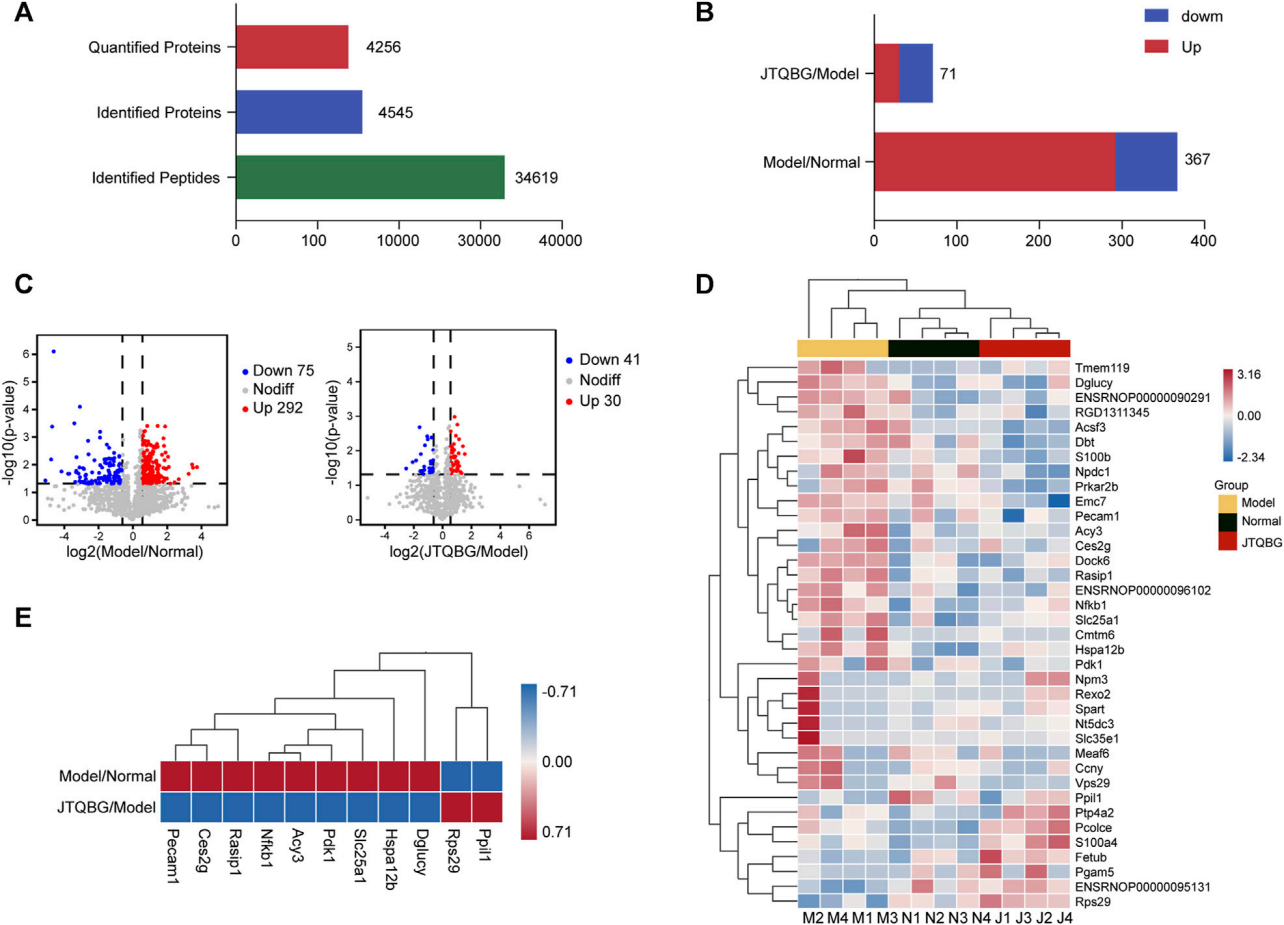
Identification of DEPs in Model/Normal and JTQBG/Model rats. **(A)** Basic statistics of the MS results. **(B)** The distribution of differentially expressed proteins (DEPs) in different comparison groups. **(C)** Volcano plot of DEPs. **(D)** Hierarchical clustering heatmap of DEPs. Changed proteins (right side) and the samples in different groups (bottom). The color from red to blue shows the relative intensity of the DEPs. **(E)** Ten DEPs in the Model/Normal group were reversed in the JTQBG/Model group. Model/Normal: Model group *versus* Normal group; JTQBG/Model: JTQBG group *versus* Model group.

A total of 34,619 peptides and 4,545 proteins were identified, and 4,256 proteins were quantitatively analysed (Figure 4A). A total of 367 DEPs (fold change >1.5, or <1/1.5, and *p* < 0.05) were identified between the Model group and the Normal group. Among them, 292 DEPs in the Model group were upregulated and 75 DEPs were downregulated compared to the Normal group. A total of 71 DEPs were changed between the JTQBG group and the Model group. Among them, 30 DEPs were upregulated, and 41 DEPs were downregulated. (Figures 4B, C). The DEPs are presented in a cluster heatmap. Interestingly, the hierarchical clustering figure showed that the synovial proteins in the Model group were significantly separated from those in the Normal group or the JTQBG group. The JTQBG group was close to the Normal group. (Figure 4D). Moreover, In Figure 4E, we aimed to illustrate the significant synovial protein changes in CIA model rats after intervention with JTQBG. To achieve this, we carefully selected the 11 proteins with the most significant differences compared to JTQBG group and Model group. Among them, nine proteins were found to be upregulated in Model group compared to the Normal group. But interestingly, they were downregulated in JTQBG group compared to Model group, including nuclear factor kappa B subunit (Nfkb1), Ras interacting protein 1 (Rasip1), D-glutamate cyclase (Dglucy), heat shock protein family A (Hspa12b), solute carrier family 25 memb (Slc25a1), aminoacylase 3 (Acy3), carboxylesterase 2G (Ces2g), pyruvate dehydrogenase kinase 1 (Pdk1), and platelet endothelial cell adhesion molecule 1 (Peacm1). Additionally, two proteins were downregulated in Model group compared to Normal group. However, they were upregulated in the JTQBG group compared to the Model group, incuding peptidylprolyl isomerase like 1 (Ppil1) and ribosomal protein S29 (Rps29).

**FIGURE 5:**
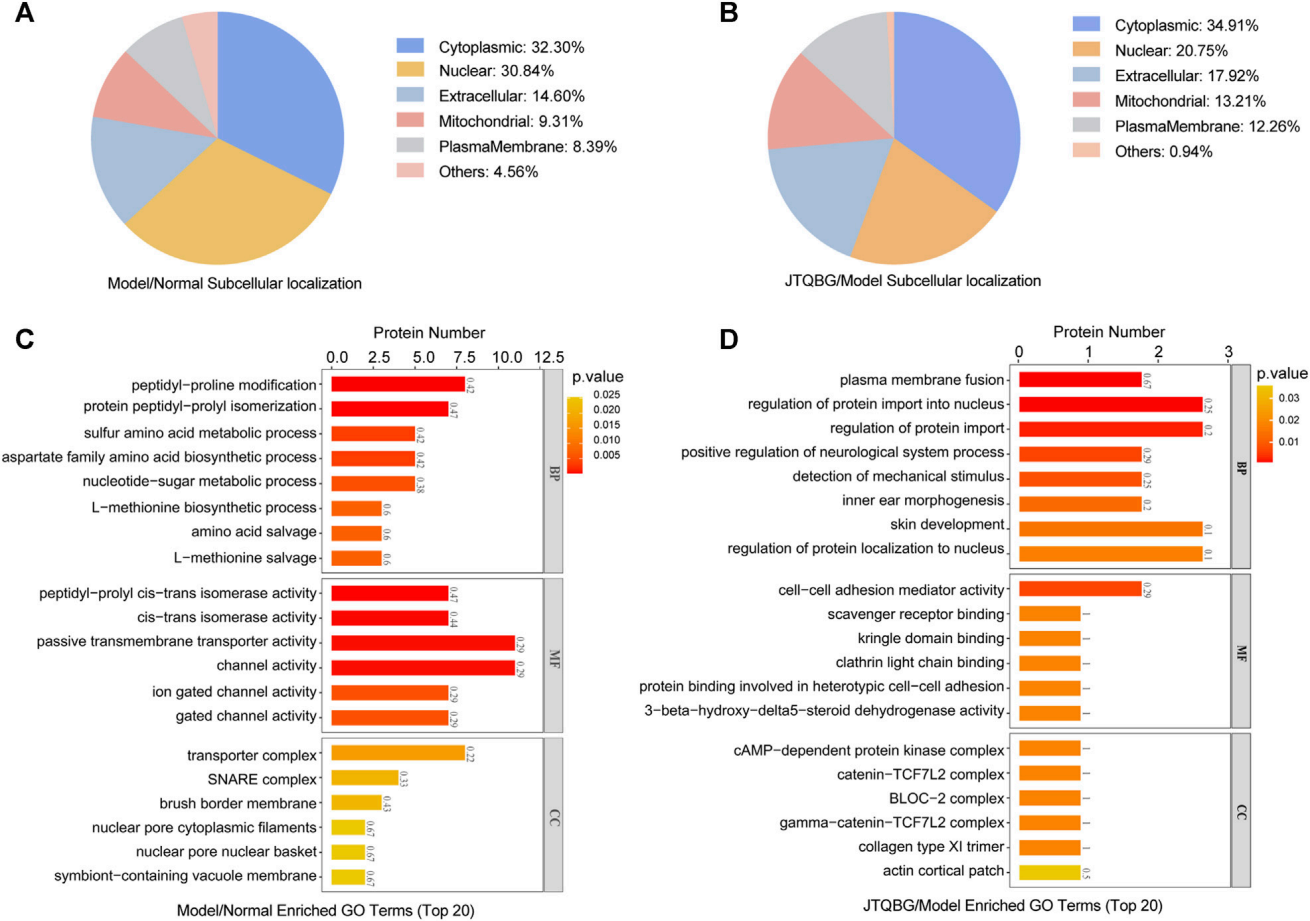
Classification of DEPs based on GO and subcellular localization. **(A and B)** Subcellular localization chart of DEPs in the Model/Normal group and JTQBG/Model group. **(C and D)** GO enrichment of DEPs in the Model/Normal group and JTQBG/Model group. The *x*-axis represents the biological process (BP), cellular component (CC), and molecular function (MF). The *y*-axis represents gene numbers.

Furthermore, subcellular localization and Gene Ontology (GO) enrichment analysis of DEPs were performed. Subcellular localization results showed that the locations of DEPs within cellular compartments in the two comparison groups (Model vs. Normal and JTQBG vs. Model) were similar. Almost one-third of DEPs were located in the cytoplasm (32.30% in Model vs. Normal, and 34.91% in JTQBG vs. Model). The rest of the DEPs were mostly located in nuclear and extracellular regions (Figures 5A, B). GO annotations included biological process (BP), cellular component (CC), and molecular function (MF). The top 20 of GO enrichment analysis are shown in Figures 5C, D. The results showed that DEPs were significantly enriched in several BP, MF and CC categories. Peptidyl-proline modification was the most enriched BP in DEPs between the Model and Normal groups, while regulation of protein import into the nucleus was the most enriched BP in DEPs between the JTQBG and Model groups. In MF terms, DEPs between the Model and Normal groups were mostly enriched in passive transmembrane transporter activity, while DEPs between the JTQBG and Model groups were enriched in cell‒cell adhesion mediator activity. In the CC category, transporter complex was the most enriched CC in DEPs between the Model and Normal groups, while cAMP-dependent protein kinase inhibitor activity was the most enriched in DEPs between the JTQBG and Model groups.

**FIGURE 6:**
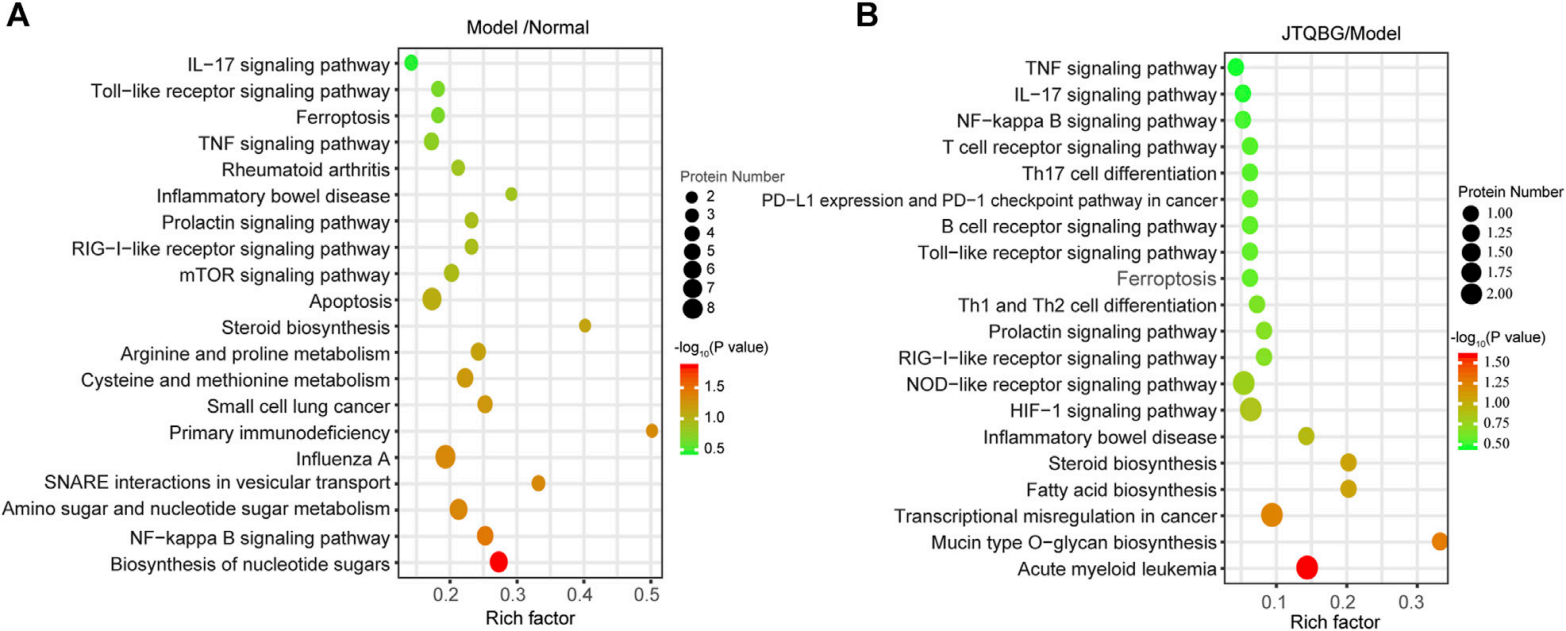
KEGG enrichment analysis and validation of NF-κB1 signaling pathway regulation. **(A and B)** KEGG pathway enrichment bubble of DEPs in the Model/Normal group and JTQBG/Model group. The circle color indicates the enrichment significance *p*-value, while the circle size indicates the number of DEPs in the KEGG pathway.

A total of 249 protein pathways were enriched between the Normal group and the Model group, while 109 protein pathways were enriched between the JTQBG group and the Model group through KEGG analysis. The DEPs between Model and Normal groups were significantly enriched in biosynthesis of nucleotide sugars, NF-kappa B signaling pathway, amino sugar and nucleotide sugar metabolism, arginine and proline metabolism, cysteine and methionine metabolism, steroid biosynthesis apoptosis, TNF signaling pathway, Toll-like receptor signaling pathway, IL-17 signaling pathway and so on (Figure 6A). The DEPs between JTQBG and Model groups were mainly enriched in mucin type O-glycan biosynthesis, fatty acid biosynthesis, steroid biosynthesis, HIF-1 signaling pathway, NOD-like receptor signaling pathway, Toll-like receptor signaling pathway, NF-kappa B signaling pathway, IL-17 signaling pathway, TNF signaling pathway and so on (Figure 6B). Interestingly, the NF-kappa B signaling pathway, closely related to the pathogenesis of RA, was enriched between the two comparison groups.

**FIGURE 7:**
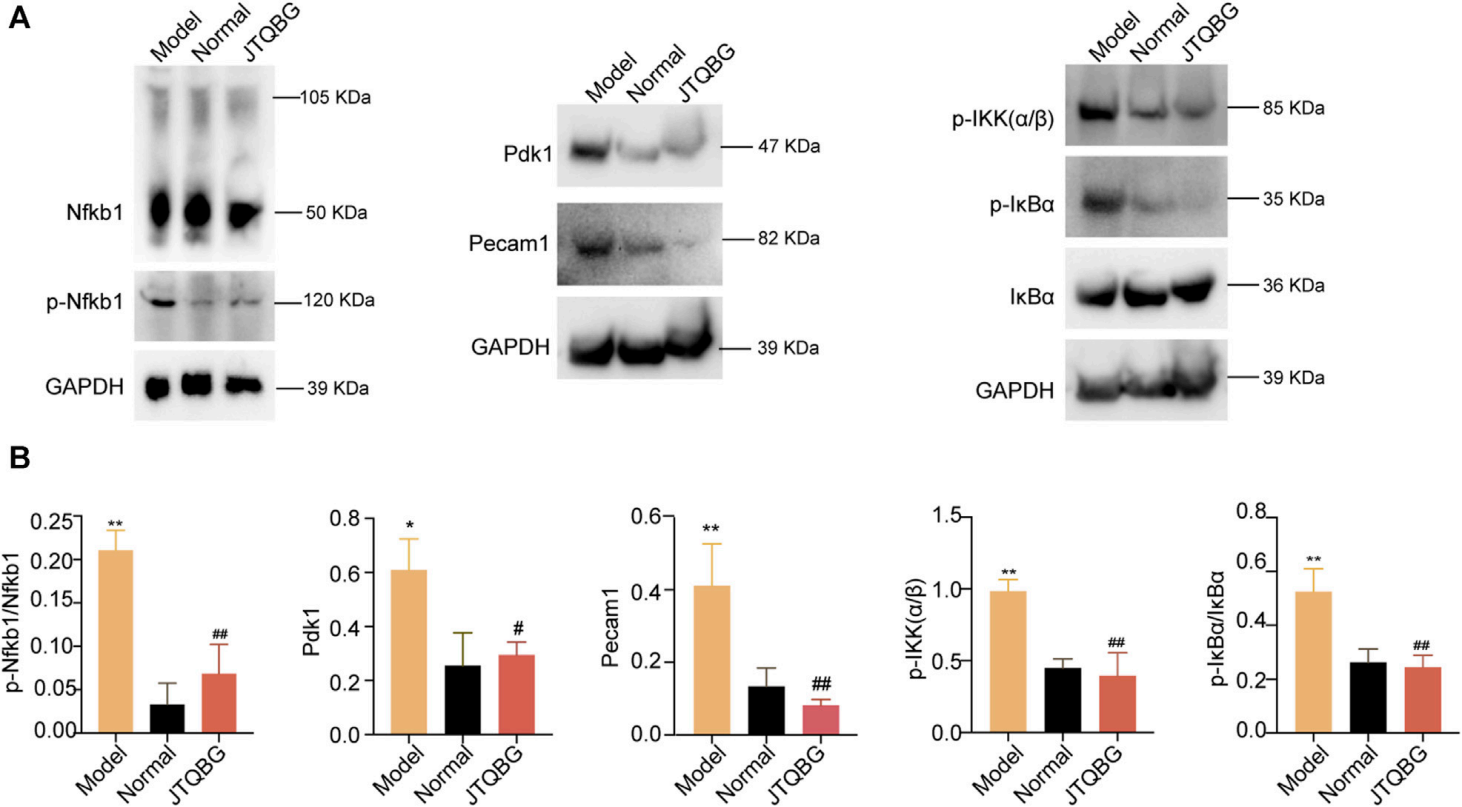
The protein modulation of Nfkb1, p-Nfkb1, Pecam1, Pdk1 and the NF-κB1 signaling pathway. **(A)** Representative Western blot images. **(B)** Semiquantitative analysis of Western blot in different groups. *p < 0.05, **p < 0.01, compared with the Normal group; #*p* < 0.05, ##*p* < 0.01, compared with the Model group.

Furthermore, we validated the key DEPs Nfkb1, Pdk1 and Pecam1 and the proteins of the NF-kappa B signaling pathway by Western blotting. The results revealed that the protein levels of phospho-Nfkb1 (p-Nfkb1), Pdk1, Pecaml, phospho-IKK(α/β) (p-IKK(α/β)) and phospho-IκBα (p-IκBα) were significantly higher in the Model group, and treatment with JTQBG reversed these expression-level alterations (Figures 7A, B).

### JTQBG partially restored serum metabolism disorders in CIA rats

The serum metabolites were analyzed using untargeted metabolomics. As shown in Supplementary Material S5, PCA showed clear separation between the Normal and Model groups, as well as between the Model and JTQBG groups. A total of 948 metabolites were identified, with 161 significantly changed metabolites (VIP >1 and *p* < 0.05, DEMs). Compared to the Normal group, 56 DEMs were upregulated (fold change >1) and 43 DEMs were downregulated in the Model group (fold change <1). Compared to the Model group, 44 DEMs were upregulated and 39 DEMs were downregulated in the JTQBG group. Metabolic pathway analysis using MetaboAnalyst 5.0 identified five common pathways related to RA that were affected by JTQBG, including Alanine, aspartate and glutamate metabolism, Ascorbate and aldarate metabolism, Arginine metabolism, Inositol phosphate metabolism, and Tryptophan metabolism. Additionally, 17 common differentially abundant metabolites were listed between the Model/Normal and JTQBG/Model groups (Supplementary Material S6). Compared to the Normal group, the serum levels of Serotonin, 2-phenylpiperidine-2-acetamide, Dl-glutamic acid, 3-[(cholamidopropyl)dimethylammonio]-1-propanesulfonate, 3-hydroxybutyrylcarnitine, 2-hydroxy-6-methylquinoline-3-carbaldehyde, Dl-lactate, and Curcumin were significantly increased in the Model group (*p* < 0.05). After JTQBG intervention, the levels of these 8 metabolites were significantly decreased (*p* < 0.05). Additionally, compared to the Normal group, the serum levels of 3-aminobutanoic acid, ornidazole, 1-octadecanoyl-2-octadecenoyl-SN-3-phosphocholine, 3-aminobutanoic acid, 1-octadecanoyl-SN-3-phosphocholine, Pc (18:1e/14, 15-eet), L-gulono-1,4-lactone, D-mannose, D-threitol, D-(+)-mannose, and D-turanose were significantly decreased in the Model group (*p* < 0.05). After JTQBG intervention, the levels of these 9 metabolites were significantly increased (*p* < 0.05).

## Discussion

Rheumatoid arthritis (RA) is a chronic autoimmune disease characterized by synovial hyperplasia, inflammatory cell infiltration, erosion of cartilage, and synovitis. However, there are currently no effective and safe drugs available for its treatment. The application of botanical drugs, particularly in Asian countries, has gained significant attention as a potential treatment for RA. JTQBG exhibits characteristics of multi-pathway and multi-target therapy for RA, making it challenging to determine its function based on chemical composition and biological activity alone. In this study, we analyzed JTQBG using UHPLC-Q Exactive Orbitrap MS, which identified a total of 49 main metabolites (Figure 1). Additionally, 12 metabolites were simultaneously and quantitatively detected.

Chlorogenic acid, isochlorogenic acid A, and galuteolin are derived from *Lonicera japonica*. Chlorogenic acid has anti-arthritic effects by inhibiting the expression of BAFF in MH7A cells through the NF-κB pathway (Fu et al., 2019). Isochlorogenic acid A suppresses TNF-α or LPS-triggered inflammatory responses by down-regulating IL-17 and the MAPK signaling pathway in MH7A and RAW264.7 cells (Yang et al., 2023). Guan et al. found that galuteolin significantly inhibits the NF-κB signaling pathway and reduces the levels of pro-inflammatory factors in TNF-α-induced RA-FLS cells (Guan et al., 2020). Sinomenine, the main metabolite of Sinomenium acutum, alleviates joint inflammation in rats with CIA by inhibiting the release of pro-inflammatory cytokines and the abnormal invasion and migration of FLSs (Lan et al., 2016; Yu et al., 2024). Paeoniflorin, the principal metabolite of Paeonia lactiflora Pall, can treat RA by reducing the levels of p-p65 and pro-inflammatory cytokines and inhibiting FLS proliferation, osteoclast differentiation, and macrophage pyroptosis (Xu et al., 2018; Xu et al., 2021; Wang et al., 2023). Ferulic acid, the main metabolite of Angelica sinensis, has anti-inflammatory and anti-arthritic properties. Ganesan and Rasool, 2019 found that ferulic acid reduces the expression of GM-CSF, RANKL, and increases the expression of OPG in AA-FLS by inhibiting the IL-17/IL-17RA/STAT-3 signaling cascade. Harpagide is the effective metabolite of Scrophularia ningpoensis Hemsl. Chung et al. found that harpagide promotes the differentiation and maturation of osteoblast cells while simultaneously suppressing the RANKL-induced differentiation of osteoclast cells (Chung et al., 2016). Monotropein is the primary metabolite of Pyrola calliantha Andres. Zhang et al. found that monotropein inhibits the differentiation of OCs and the expression of the NF-κB and Akt/GSK-3β signaling pathway in OCs derived from BMMs (Zhang et al., 2022). Liquiritin, a natural flavonoid extracted from the roots of Glycyrrhiza glabra, suppresses inflammatory infiltration, angiogenesis, synovial hyperplasia, and the proliferation of IL-1β-induced RA-FLS (Zhai et al., 2019). The anti-inflammatory effects of these metabolites support the anti-inflammatory effects of JTQBG. Further details can be found in Supplementary Material S7. Although we have identified the main metabolites of the JTQBG, it is difficult for us to conduct pharmacological research on each individual metabolite due to the large number of them. We have only discussed the roles played by the above-mentioned main metabolites through literature review. This is also a limitation of our experiment, and further research will be necessary to explore the specific bioactive metabolites of JTQBG through network pharmacology and molecular docking.

Moreover, we investigated the intervention effect of JTQBG on CIA rats and explored the underlying mechanisms using synovial proteomics technology. Our results showed that JTQBG could exert anti-RA effects by inhibiting inflammation, the immune response, synovial hyperplasia and cartilage damage. In addition, the 4D label-free proteomics analysis of synovial tissues demonstrated that multiple DEPs and enriched signaling pathways probably participated in the molecular mechanisms of JTQBG. On the one hand, we found that JTQBG treatment significantly improved the arthritis index, paw redness and swelling. Ultrasound examination found that JTQBG could significantly inhibit synovial hyperplasia, joint inflammation and arthroedema. The therapeutic efficacy was further confirmed by joint histopathological examination, which revealed that JTQBG could inhibit synovial proliferation, inflammatory cell infiltration, pannus formation, and bone and cartilage erosion. The results of Saffron-O and fast green staining showed that JTQBG could reduce the degradation of cartilage matrix and cartilage damage. As a chronic autoimmune disease, RA can lead to the proliferation of spleen cells, white pulp hyperplasia and splenomegaly. Our research demonstrated that JTQBG decreased the spleen index and inhibited the proliferation of white pulp in CIA rats, suggesting that JTQBG had an immunosuppressive effect. Pro-inflammatory cytokines, such as IL-1β and IL-18, are crucial in the progression of RA. It is well-known that IL-1β acts as a key mediator in activating the NF-κB signaling pathway, which subsequently stimulates the proliferation of synovial fibroblasts and leads to synovitis (Migliorini et al., 2020). Similarly, IL-18, a member of the IL-1 family, shares similarities with IL-1β and also activates the NF-κB signaling pathway, resulting in the production of inflammatory mediators (Kaplanski, 2018). Furthermore, IL-18 can induce the expression of TNF-α, IL-6, and IFN-γ, leading to joint inflammation and cartilage erosion (Nozaki et al., 2019). The activation of NF-κB promotes the transcription of NLRP3, pro-IL-1β, and pro-IL-18, ultimately leading to caspase-1 activation, pyroptosis, and the extensive release of IL-1β and IL-18 (Xu et al., 2021). Our findings demonstrate that treatment with JTQBG significantly reduces the levels of IL-1β and IL-18, suggesting its potential involvement in regulating the NF-κB signaling pathway in synovitis. Taken together, JTQBG is an effective traditional Chinese medicine formulation for the treatment of RA.

Furthermore, to explore the potential biological mechanisms in synovitis and synovial hyperplasia, we conducted 4D label-free proteomic experiments to compare protein profiles among the Model, Normal and JTQBG groups. A total of 4256 proteins were quantifiable in all three groups, with 367 DEPs between the Model and Normal groups, as well as 71 DEPs between the JTQBG and Model groups. Moreover, eleven DEPs were significantly reversed after treatment with JTQBG, such as Nfkb1, Pdk-1 and Pecam1. Subsequently, subcellular localization and GO category analysis showed that the regulation of protein import into the nucleus, cell‒cell adhesion mediator activity, and cAMP-dependent protein kinase inhibitor activity might be linked to the regulatory roles of JTQBG in RA. Notably, KEGG enrichment pathway analysis demonstrated that the NF-κB signaling pathway was significantly enriched in DEPs between the Model and Normal groups, as well as the JTQBG and Model groups. The NF-κB signaling pathway modulates several physiological processes of inflammation, immunity and apoptosis. It stimulates the secretion of inflammatory factors, causing excessive immune and inflammatory responses in the pathological process of RA (Xu et al., 2009; Liu et al., 2017). Overexpression of the NF-κB signaling pathway has been noted in the synovial tissues of patients with RA. Therefore, inhibition of the NF-κB signaling pathway might be a key point in RA therapy. To clarify the pharmacological mechanism of JTQBG in RA, the expression levels of NF-κB signaling molecules were detected by Western blotting. The results demonstrated that both p-IκBα and p-IKKα/β were significantly downregulated. JTQBG could inhibit activation of the NF-κB signaling pathway, thereby inhibiting the progression of RA.

To further validate the proteomics results, the key DEPs were analysed by Western blotting. Our results showed that JTQBG treatment significantly reduced the expression of p-Nfkb1, Pdk1 and Peacm1. Nfkb1 (p50), a member of the NF-κB signaling pathway family, is an important transcription factor for the pathogenesis of RA (Duan and Li, 2018). The success rate of the CIA model was significantly reduced in Nfkb1-deficient mice (Campbell et al., 2000). In addition, Nfkb1 could inhibit cartilage degradation and joint destruction by affecting MMP gene transcription (Vincenti et al., 1998). Of note, a total of 73 signaling pathways were associated with Nfkb1 (Supplementary Material S8). Of them, we found six inflammation-associated pathways, eight pathways related to immunological regulation and other pathways associated with cell proliferation/apoptosis and bone destruction. Overall, the differential expression in these signaling pathways caused by the differential expression of Nfkb1 has provided us with some hints for the potential biological mechanisms of JTQBG treatment. Pdk1 was associated with the proliferation and apoptosis of several cell lines (Zeng et al., 2002; Han et al., 2014). Pdk1 was highly expressed in the RA synovial tissue. Overexpression of Pdk1 could increase the invasiveness of MH7A cells and the secretion of IL-1β and IL-6 (Sun et al., 2017). Pecam1, known as CD31, is a key factor in platelet adhesion and aggregation (Gao et al., 2005). The expression of Pecam1 was increased in RA synovial tissue (Cao et al., 2022). The inhibition of Pecam1 could significantly decrease paw swelling, joint symptom score and inflammatory cell infiltration in CIA mice (Ishikaw et al., 2002). Overall, our study found that JTQBG could be used to treat RA, probably by inhibiting the expression of Nfkb1, Pdk1, Peacm1 and the NF-κB signaling pathway.

Finally, we investigated the changes in serum metabolites in CIA rats after the intervention of JTQBG. Metabolic profiling of CIA rats treated with JTQBG revealed significant changes in a total of 17 metabolites. These metabolites were closely associated with Alanine, aspartate, and glutamate metabolism, Ascorbate and aldarate metabolism, and Tryptophan metabolism. Amino acids play a crucial role in modulating the immune response and exhibit significant metabolic abnormalities in both CIA rats and RA patients (Li et al., 2018; He et al., 2019). Narasimhan et al. discovered that abnormal metabolism of Alanine, aspartic acid, and glutamate was linked to the expression of the inflammatory cytokine TNF-α in the synovium (Narasimhan et al., 2018). Previous studies demonstrated that Serotonin, a key metabolite in Tryptophan metabolism, could induce joint inflammation and pain in a mouse model of arthritis, while Serotonin receptor antagonists attenuated the severity of arthritis (Fakhfouri et al., 2012; Chabbi-Achengli et al., 2016), confirming the relationship between serotonin and RA severity. In our study, we observed disturbances in Alanine, aspartate, and glutamate metabolism in CIA rats, characterized by decreased levels of Glutamine and increased levels of Glutamate and Aspartic acid. Additionally, the level of Serotonin significantly increased in CIA rats. After JTQBG intervention, these metabolites partially recovered, suggesting a regulatory effect of JTQBG on the disrupted Alanine, aspartate, and glutamate metabolism, as well as Tryptophan metabolism. Furthermore, Ascorbate and aldarate metabolism have been implicated in various diseases, including cancer and ulcerative colitis. A previous study has demonstrated that Ascorbate and aldarate metabolism was significantly upregulated in the liver of a breast cancer mouse model induced by PBdes, which impaired cellular reducing ability, induced oxidative stress, and aggravated cancer progression (Wei et al., 2020). In our study, we observed disturbances in Ascorbate and aldarate metabolism in CIA rats, which showed partial recovery after JTQBG intervention, suggesting their potential involvement in the pathogenesis of RA.

Although this study demonstrated that JTQBG is effective in the reatment of RA, there are still some limitations. First, JTQBG has the characteristics of multi-target and multi-pathway action. In this study, we only investigate the regulatory effects of JTQBG on NF-κB signaling pathway, though JTQBG also have certain regulatory effects on TNF signaling pathway, IL-17 signaling pathway as well as some metabolic pathways, such as steroid biosynthesis and fatty acid biosynthesis (Figure 6B). Studies have demonstrated that the metabolic pathways of RA patients and CIA models were altered, and some metabolic pathways might involved in the pathogenesis of RA by promoting inflammation and immune response (Duarte-Delgado et al., 2022; Takahashi et al., 2017; Ding et al., 2023; Kim et al., 2014; Young et al., 2013). However, one of the limitations in this study is that we do not further investigate the roles these metabolic pathways in the therapeutic mechanisms of JTQBG. Secondly, we only conduct X-ray examination for evaluating the joint damage. In the further study, we will conduct CT or MRI examination to evaluate the effect of JTQBG on preventing joint damage.

## Conclusion

In conclusion, our study demonstrated that JTQBG effectively improved joint and systemic inflammation, immune responses, synovial hyperplasia, and bone and cartilage damage *in vivo*. The anti-arthritic mechanism of JTQBG may be attributed to its inhibition of the NF-κB signaling pathway and its impact on serum metabolites. Our findings provide valuable insights into the pharmacological mechanism of JTQBG and offer valuable guidance for its rational clinical application in the treatment of RA.

## Data Availability

The datasets presented in this study can be found in online repositories and supplementary materials. The data have been deposited to the ProteomeXchange Consortium (http://proteomecentral.proteomexchange.org) via the iProX partner repository with the accession number PXD050056.
