# Supplementary material for: The protective effect of traditional Chinese medicine Jinteng Qingbi granules on rats with rheumatoid arthritis
Source: Front Pharmacol. 2024 Mar 13;15:1327647. doi: 10.3389/fphar.2024.1327647 (PMC10965689; doi:10.3389/fphar.2024.1327647)
Supplement: Supplementary file 1 [file DataSheet4.docx]

**
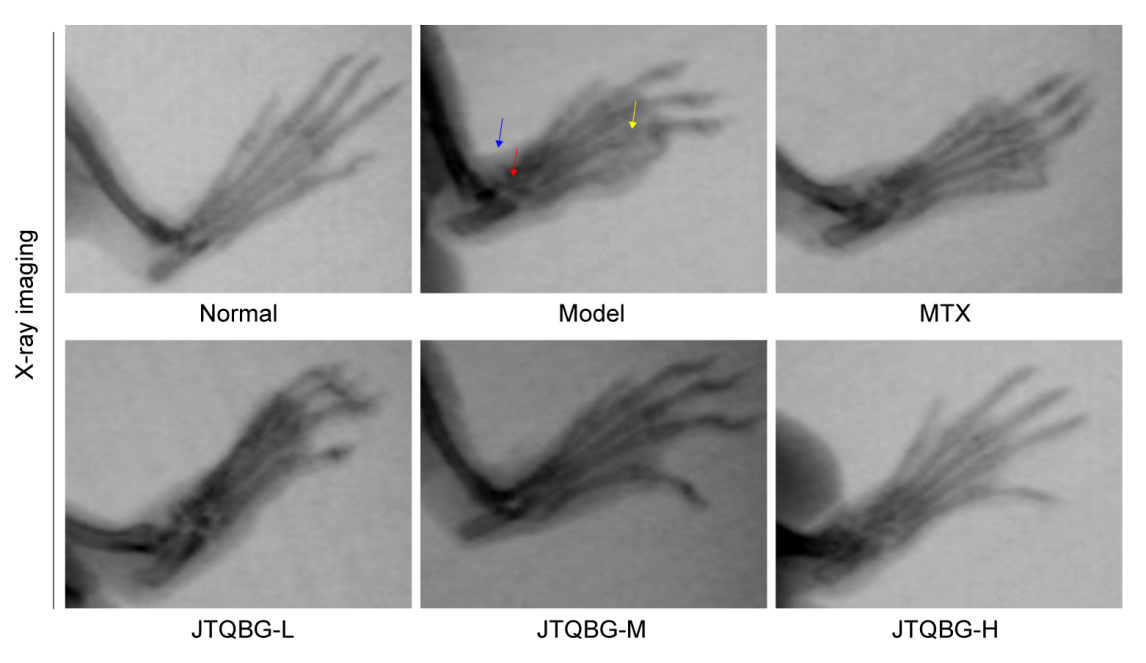
**

**Supplementary material 4:** Effects of JTQBG on the joint destruction of CIA rats. Representative images of paw joints X-ray imaging. Articular soft tissue swelling was marked by blue arrows. Joint space narrows was marked by red arrows. Bone erosion was marked by yellow arrows.
