# Supplementary material for: The protective effect of traditional Chinese medicine Jinteng Qingbi granules on rats with rheumatoid arthritis
Source: Front Pharmacol. 2024 Mar 13;15:1327647. doi: 10.3389/fphar.2024.1327647 (PMC10965689; doi:10.3389/fphar.2024.1327647)
Supplement: Supplementary file 2 [file DataSheet7.docx]

**Supplementary material 7:** The mechanism of the main metabolites of JTQBG in treating RA

| Chinese medicine | main metabolites | content | Mechanism and effect |
| --- | --- | --- | --- |
| Lonicera japonica Thunb. | chlorogenic acid | 294.27 μg/g | Inhibit the expression of BAFF in MH7A cells through NF-κB pathway (Fu et al., 2019) |
|  | isochlorogenic acid A | 205.40 μg/g | Suppress TNF-α or LPS-triggered inflammatory responses via down-regulating IL-17 signaling and the MAPK pathway in MH7A cells and in RAW264.7 cells, prevent osteoclasts from differentiating (Yang et al., 2023) |
|  | galuteolin | 40.00 μg/g | Inhibit the levels of Iκ-κβ, p-p65, and p-IκB, and reduced the levels of pro-inflammatory factors IL-1β, IL-6, IL-8, and MMP-1 in TNF-α-induced RA-FLS (Guan et al., 2020) |
| Paeonia lactiflora Pall. | paeoniflori | 1018.12μg/g | Inhibit abnormal proliferation and migration of FLS by regulating NF-κB signaling, regulate the M1/M2 ratio, decrease the ratio of macrophage pyroptosis and the expressions of TLR4, MyD88, NLRP3, Caspase-1, ASC, and GSDMD-N.(Xu et al., 2021; Wang et al., 2023; Xu et al., 2018) |
| Sinomenium acutum (Thunb.) Rehd. et wils. | sinomenine | 560.08 μg/g | Modulate CRMP2 T514 phosphorylation and its nuclear translocation of FLSs, inhibit pro-inflammatory cytokine release, and suppress abnormal invasion and migration (Yu et al., 2024) |
| Angelica sinensis (Oliv.) Diels | ferulic acid | 121.60 μg/g | Inhibit the IL-17-mediated expression of TLR-3, Cyr61, IL-23, GM-CSF, RANKL, and increase the expression of OPG in AA-FLS via the inhibition of IL-17/IL-17RA/STAT-3 signaling cascade (Ganesan et al., 2019) |
| Pyrola calliantha H. Andres | monotropein | 111.00 μg/g | Reduce the number of TRAP positive OCs, inhibit the formation, differentiation, and F-actin ring construction of OCs, and inhibit the expression of the NFATc1, C-Fos, MMP-9, CtsK, TRAP, p-Akt, GSK-3β and NF-κB pathway in OCs derived from BMMs (Zhang et al., 2022) |
| *Scrophularia ningpoensis* Hemsl. | harpagide | 94.21 μg/g | Stimulate the process of differentiation and maturation of osteoblast cells, suppress the process of RANKL-induced differentiation of osteoclast cells (Chung et al., 2016) |
| Glycyrrhiza glabra L. | liquiritin | 90.44 μg/g | Inhibit the proliferation and promote cell apoptosis of IL-1β-induced-RA-FLS, down-regulate the ratio of Bcl-2/Bax and inhibit the expression of VEGF, p-JNK and p-P38 (Zhai et al., 2019) |
| Rehmannia glutinosa Libosch. | rehmannioside D | 68.66 μg/g | - |
| Cremastra appendiculata (D.Don) Makino | betaine | 34.67 μg/g | Inhibit aberrant angiogenesis in subchondral bone, inhibit osteoclastogenesis in vitro by reducing ROS production and subsequent MAPK signaling (Yajun et al., 2021) |
| Scleromitrion diffusum (Willd.) R.J.Wang | quercetin | 4.94 μg/g | Remove excess ROS, avoid cell apoptosis, and inhibit the polarization of inflammatory macrophages by reducing the activation of the NF-κB pathways (Han et al., 2023) |
