# Supplementary material for: The protective effect of traditional Chinese medicine Jinteng Qingbi granules on rats with rheumatoid arthritis
Source: Front Pharmacol. 2024 Mar 13;15:1327647. doi: 10.3389/fphar.2024.1327647 (PMC10965689; doi:10.3389/fphar.2024.1327647)
Supplement: Supplementary file 3 [file DataSheet6.docx]

**Supplementary material 6:** The common differential metabolites of between Model/Normal group and JTQBG/Model group.

| Metabolites | Adduct | Measured(m/z) | Retention(min) | Metabolic pathway | VIP | | P-value | |
| --- | --- | --- | --- | --- | --- | --- | --- | --- |
|  |  |  |  |  | M vs N | J vs M | M vs N | J vs M |
| Serotonin | [M+H-NH3]+ | 160.07 | 232.73 | Tryptophan metabolism | 5.45 | 2.35 | ↑** | ↓* |
| 2-phenylpiperidine-2-acetamide | [M+H]+ | 219.13 | 360.62 | _ | 1.49 | 1.26 | ↑** | ↓** |
| Ornidazole | [M+H]+ | 220.06 | 25.22 | _ | 1.12 | 1.12 | ↓** | ↑** |
| DL-Glutamic acid | [M+H]+ | 148.06 | 389.68 | Alanine, aspartate and glutamate metabolism | 2.17 | 2.07 | ↑** | ↓* |
| 3-[(cholamidopropyl)dimethylammonio]-1-propanesulfonate | [M+H-C5H13SO3N]+ | 448.34 | 160.87 | C11321 | 1.95 | 1.73 | ↑** | ↓** |
| 3-Aminobutanoic acid | (M+H-H2O)+ | 86.06 | 110.63 | HMDB0031654 | 1.25 | 1.23 | ↓** | ↑** |
| 1-octadecanoyl-2-octadecenoyl-sn-glycero-3-phosphocholine | [M+Na]+ | 810.60 | 133.11 | _ | 24.39 | 24.00 | ↓* | ↑** |
| 3-hydroxybutyrylcarnitine | [M+H]+ | 248.15 | 311.44 | HMDB0062735 | 2.53 | 3.26 | ↑* | ↓** |
| 2-hydroxy-6-methylquinoline-3-carbaldehyde | [M-H]- | 186.05 | 219.68 | _ | 6.16 | 5.10 | ↑** | ↓** |
| Dl-lactate | [M-H]- | 89.03 | 219.64 | C01432 | 14.11 | 12.92 | ↑** | ↓** |
| Pc(18:1e/14,15-eet) | [M+Hac-H]- | 868.61 | 132.21 | _ | 3.10 | 5.67 | ↓** | ↑* |
| L-gulono-1,4-lactone | [M-H]- | 177.04 | 102.52 | Ascorbate and aldarate metabolism | 3.37 | 4.80 | ↓** | ↑** |
| D-Mannose | (M+CH3COO)- | 239.08 | 295.66 | Galactose metabolism | 2.58 | 3.61 | ↓** | ↑** |
| D-Threitol | (M+Na-2H)- | 143.03 | 296.03 | C16884 | 1.08 | 1.54 | ↓** | ↑** |
| D-(+)-mannose | [M-H]- | 179.06 | 296.02 | C00936 | 10.10 | 14.28 | ↓** | ↑** |
| D-turanose | [M-H-C6H12O6]- | 161.05 | 295.98 | C19636 | 1.85 | 2.79 | ↓* | ↑** |
| Curcumin | [M-H]- | 367.11 | 298.47 | C10443 | 9.12 | 4.46 | ↑* | ↓* |

ANOVA technique was used to evaluate the data. *P<0.05，* *P<0.01
