# Supplementary material for: The protective effect of traditional Chinese medicine Jinteng Qingbi granules on rats with rheumatoid arthritis
Source: Front Pharmacol. 2024 Mar 13;15:1327647. doi: 10.3389/fphar.2024.1327647 (PMC10965689; doi:10.3389/fphar.2024.1327647)
Supplement: Supplementary file 4 [file DataSheet3.docx]

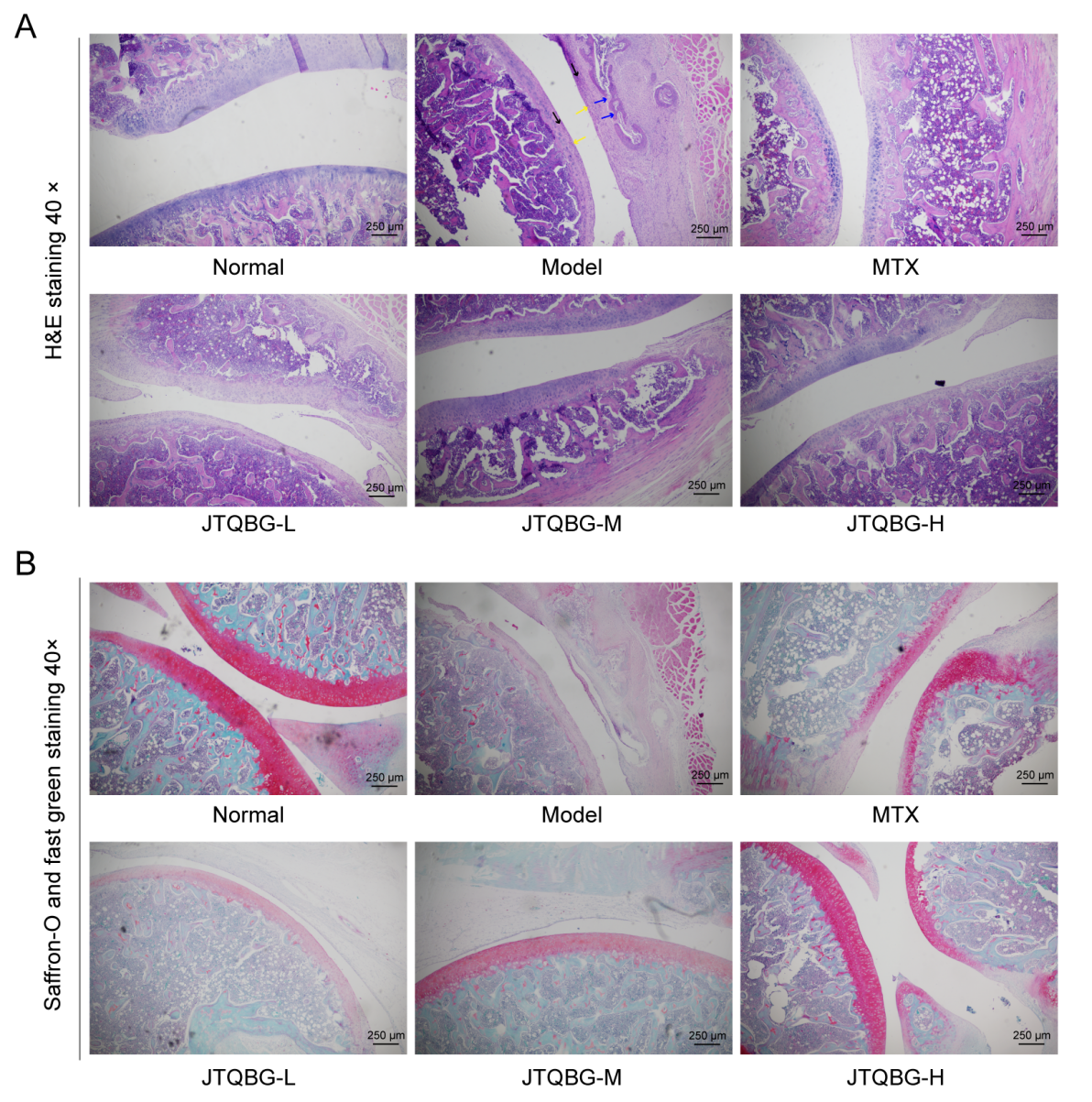


**Supplementary material 3:** Lower magnification image of H&E staining and Saffron-O and fast green staining. (A) Representative images of H&E staining of knee joints. Scale bar: 250 µm. Inflammatory cells were marked by black arrows. Synovial hyperplasia was marked by yellow arrows. Cartilage erosion was marked by blue arrows. (B) Representative images of Saffron-O and fast green staining of knee joints. Scale bar: 250 µm.
