# Supplementary material for: The protective effect of traditional Chinese medicine Jinteng Qingbi granules on rats with rheumatoid arthritis
Source: Front Pharmacol. 2024 Mar 13;15:1327647. doi: 10.3389/fphar.2024.1327647 (PMC10965689; doi:10.3389/fphar.2024.1327647)
Supplement: Supplementary file 5 [file DataSheet5.docx]

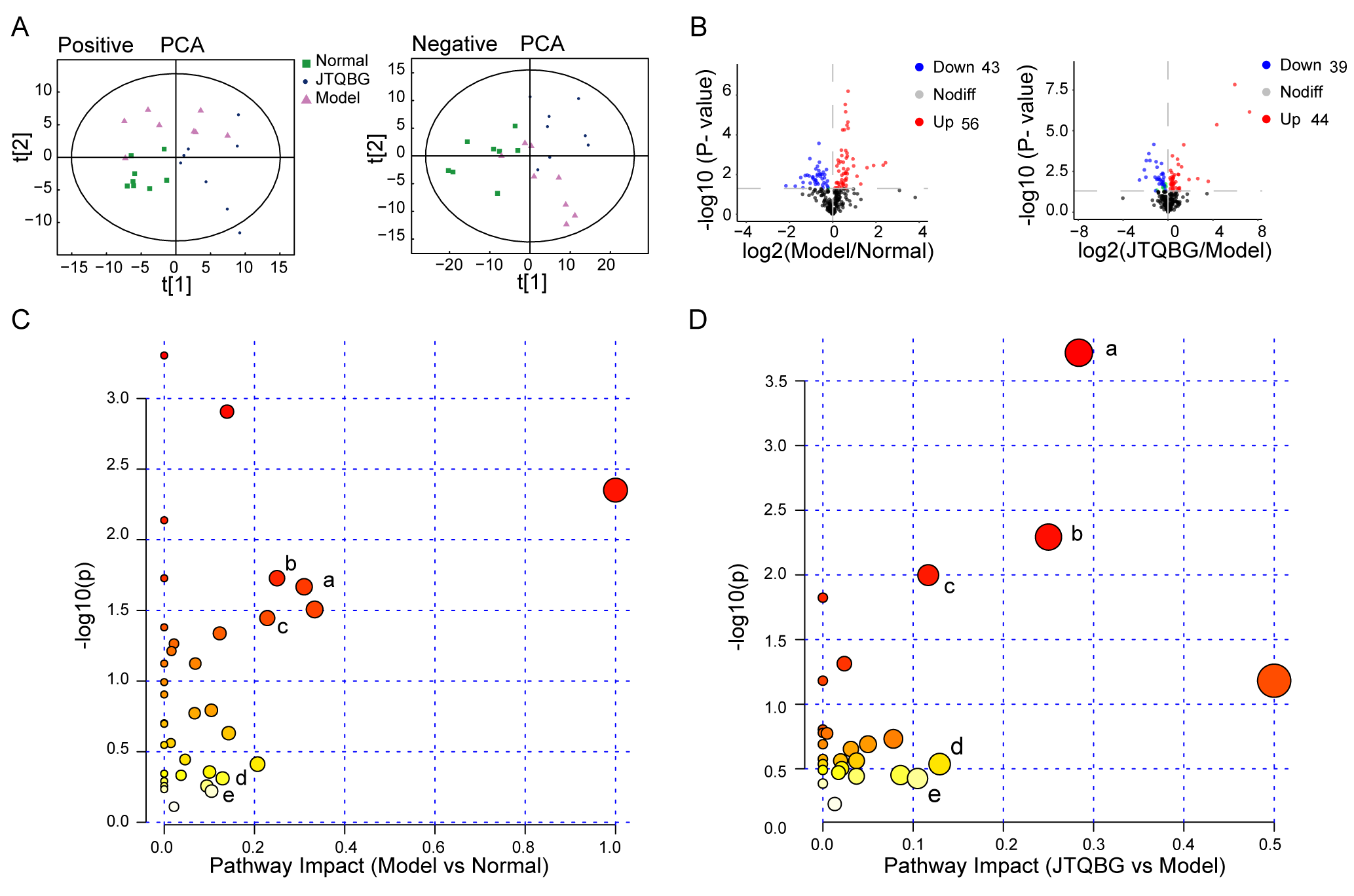


**Supplementary material 5:** Effects of JTQBG on the serum metabolites of CIA rats. (A) PCA analysis of metabolites in the three group in positive and negative ion mode. (B) Volcanic map analysis of differential metabolites between the Model group and the Normal group, the JTQBG group and the Model group. (C) Bubble map of metabolic pathway of differential metabolite enrichment between the Model group and Normal group, the JTQBG group and Model group. a: Alanine, aspartate and glutamate metabolism, b: Ascorbate and aldarate metabolism, c: Arginine metabolism, d: Inositol phosphate metabolism, e: tryptophan metabolism.
