# Supplementary material for: The protective effect of traditional Chinese medicine Jinteng Qingbi granules on rats with rheumatoid arthritis
Source: Front Pharmacol. 2024 Mar 13;15:1327647. doi: 10.3389/fphar.2024.1327647 (PMC10965689; doi:10.3389/fphar.2024.1327647)
Supplement: Supplementary file 6 [file DataSheet8.docx]

**Supplementary material 8:** 73 signaling pathways associated with NF-κB1

| Map name | Test_per | Ref_per | P value | FDR | Rich Factor |
| --- | --- | --- | --- | --- | --- |
| Pathways of neurodegeneration - multiple diseases | 0.05 | 0.04 | 0.39 | 0.62 | 0.02 |
| MicroRNAs in cancer | 0.01 | 0.01 | 0.48 | 0.62 | 0.03 |
| Pancreatic cancer | 0.01 | 0.00 | 0.24 | 0.62 | 0.07 |
| Pertussis | 0.01 | 0.01 | 0.39 | 0.62 | 0.04 |
| Small cell lung cancer | 0.01 | 0.00 | 0.25 | 0.62 | 0.06 |
| Human papillomavirus infection | 0.01 | 0.02 | 0.73 | 0.73 | 0.01 |
| Human immunodeficiency virus 1 infection | 0.01 | 0.01 | 0.62 | 0.64 | 0.02 |
| Sphingolipid signaling pathway | 0.01 | 0.01 | 0.45 | 0.62 | 0.03 |
| Adipocytokine signaling pathway | 0.01 | 0.00 | 0.27 | 0.62 | 0.06 |
| Antifolate resistance | 0.01 | 0.00 | 0.17 | 0.62 | 0.10 |
| Yersinia infection | 0.01 | 0.01 | 0.47 | 0.62 | 0.03 |
| Hepatitis B | 0.01 | 0.01 | 0.41 | 0.62 | 0.03 |
| T cell receptor signaling pathway | 0.01 | 0.00 | 0.28 | 0.62 | 0.06 |
| IL-17 signaling pathway | 0.01 | 0.00 | 0.33 | 0.62 | 0.05 |
| Lipid and atherosclerosis | 0.01 | 0.01 | 0.67 | 0.69 | 0.02 |
| Human cytomegalovirus infection | 0.01 | 0.01 | 0.60 | 0.62 | 0.02 |
| Chemical carcinogenesis - reactive oxygen species | 0.04 | 0.02 | 0.24 | 0.62 | 0.03 |
| Alcoholic liver disease | 0.01 | 0.01 | 0.42 | 0.62 | 0.03 |
| Kaposi sarcoma-associated herpesvirus infection | 0.01 | 0.01 | 0.57 | 0.62 | 0.02 |
| Relaxin signaling pathway | 0.01 | 0.01 | 0.40 | 0.62 | 0.04 |
| RIG-I-like receptor signaling pathway | 0.01 | 0.00 | 0.21 | 0.62 | 0.08 |
| Prostate cancer | 0.01 | 0.00 | 0.25 | 0.62 | 0.06 |
| Measles | 0.01 | 0.01 | 0.38 | 0.62 | 0.04 |
| HIF-1 signaling pathway | 0.02 | 0.01 | 0.14 | 0.62 | 0.06 |
| Inflammatory bowel disease | 0.01 | 0.00 | 0.12 | 0.62 | 0.14 |
| NOD-like receptor signaling pathway | 0.02 | 0.01 | 0.16 | 0.62 | 0.05 |
| Alzheimer disease | 0.05 | 0.03 | 0.28 | 0.62 | 0.03 |
| Toll-like receptor signaling pathway | 0.01 | 0.00 | 0.27 | 0.62 | 0.06 |
| Cocaine addiction | 0.01 | 0.00 | 0.10 | 0.62 | 0.17 |
| Pathways in cancer | 0.02 | 0.02 | 0.51 | 0.62 | 0.02 |
| Insulin resistance | 0.01 | 0.01 | 0.40 | 0.62 | 0.04 |
| Epstein-Barr virus infection | 0.01 | 0.01 | 0.59 | 0.62 | 0.02 |
| Hepatitis C | 0.01 | 0.01 | 0.45 | 0.62 | 0.03 |
| Herpes simplex virus 1 infection | 0.01 | 0.01 | 0.52 | 0.62 | 0.03 |
| Chemical carcinogenesis - receptor activation | 0.01 | 0.01 | 0.43 | 0.62 | 0.03 |
| NF-kappa B signaling pathway | 0.01 | 0.00 | 0.31 | 0.62 | 0.05 |
| Chemokine signaling pathway | 0.01 | 0.01 | 0.49 | 0.62 | 0.03 |
| Human T-cell leukemia virus 1 infection | 0.01 | 0.01 | 0.53 | 0.62 | 0.02 |
| PI3K-Akt signaling pathway | 0.01 | 0.01 | 0.71 | 0.72 | 0.01 |
| C-type lectin receptor signaling pathway | 0.01 | 0.01 | 0.37 | 0.62 | 0.04 |
| Chronic myeloid leukemia | 0.01 | 0.00 | 0.21 | 0.62 | 0.08 |
| Diabetic cardiomyopathy | 0.04 | 0.02 | 0.30 | 0.62 | 0.03 |
| Osteoclast differentiation | 0.01 | 0.01 | 0.37 | 0.62 | 0.04 |
| B cell receptor signaling pathway | 0.01 | 0.00 | 0.27 | 0.62 | 0.06 |
| Prolactin signaling pathway | 0.01 | 0.00 | 0.21 | 0.62 | 0.08 |
| Non-alcoholic fatty liver disease | 0.04 | 0.01 | 0.10 | 0.62 | 0.05 |
| Coronavirus disease - COVID-19 | 0.02 | 0.02 | 0.41 | 0.62 | 0.03 |
| Acute myeloid leukemia | 0.02 | 0.00 | 0.03 | 0.62 | 0.14 |
| Viral carcinogenesis | 0.01 | 0.01 | 0.59 | 0.62 | 0.02 |
| Longevity regulating pathway | 0.01 | 0.00 | 0.23 | 0.62 | 0.07 |
| Legionellosis | 0.01 | 0.00 | 0.33 | 0.62 | 0.05 |
| Neutrophil extracellular trap formation | 0.01 | 0.01 | 0.50 | 0.62 | 0.03 |
| PD-L1 expression and PD-1 checkpoint pathway in cancer | 0.01 | 0.00 | 0.27 | 0.62 | 0.06 |
| Cellular senescence | 0.01 | 0.01 | 0.47 | 0.62 | 0.03 |
| Transcriptional misregulation in cancer | 0.02 | 0.00 | 0.06 | 0.62 | 0.09 |
| Influenza A | 0.01 | 0.01 | 0.54 | 0.62 | 0.02 |
| Amoebiasis | 0.01 | 0.01 | 0.46 | 0.62 | 0.03 |
| Th1 and Th2 cell differentiation | 0.01 | 0.00 | 0.23 | 0.62 | 0.07 |
| Cytosolic DNA-sensing pathway | 0.01 | 0.00 | 0.14 | 0.62 | 0.13 |
| TNF signaling pathway | 0.01 | 0.01 | 0.34 | 0.62 | 0.04 |
| Neurotrophin signaling pathway | 0.01 | 0.01 | 0.38 | 0.62 | 0.04 |
| Apoptosis | 0.01 | 0.01 | 0.53 | 0.62 | 0.02 |
| Toxoplasmosis | 0.01 | 0.01 | 0.39 | 0.62 | 0.04 |
| Tuberculosis | 0.01 | 0.01 | 0.56 | 0.62 | 0.02 |
| Th17 cell differentiation | 0.01 | 0.00 | 0.28 | 0.62 | 0.06 |
| Chagas disease | 0.01 | 0.00 | 0.33 | 0.62 | 0.05 |
| MAPK signaling pathway | 0.01 | 0.01 | 0.59 | 0.62 | 0.02 |
| Ras signaling pathway | 0.01 | 0.01 | 0.48 | 0.62 | 0.03 |
| Fluid shear stress and atherosclerosis | 0.02 | 0.01 | 0.19 | 0.62 | 0.05 |
| cAMP signaling pathway | 0.01 | 0.01 | 0.52 | 0.62 | 0.03 |
| AGE-RAGE signaling pathway in diabetic complications | 0.01 | 0.01 | 0.42 | 0.62 | 0.03 |
| Salmonella infection | 0.02 | 0.02 | 0.53 | 0.62 | 0.02 |
| Leishmaniasis | 0.01 | 0.00 | 0.25 | 0.62 | 0.06 |
